# Mismatch Negativity Unveils Tone Perception Strategies and Degrees of Tone Merging: The Case of Macau Cantonese

**DOI:** 10.3390/brainsci14121271

**Published:** 2024-12-17

**Authors:** Han Wang, Fei Gao, Jingwei Zhang

**Affiliations:** 1 Faculty of Arts and Humanities, University of Macau, Macau SAR 999078, China; yc37729@um.edu.mo; 2 Centre for Cognitive and Brain Sciences, University of Macau, Macau SAR 999078, China; 3 Institute of Modern Languages and Linguistics, Fudan University, Shanghai 200433, China

**Keywords:** Cantonese, tone merging, language variation, cognitive function, EEG, MMN

## Abstract

Background/Objectives: Previous studies have examined the role of working memory in cognitive tasks such as syntactic, semantic, and phonological processing, thereby contributing to our understanding of linguistic information management and retrieval. However, the real-time processing of phonological information—particularly in relation to suprasegmental features like tone, where its contour represents a time-varying signal—remains a relatively underexplored area within the framework of Information Processing Theory (IPT). This study aimed to address this gap by investigating the real-time processing of similar tonal information by native Cantonese speakers, thereby providing a deeper understanding of how IPT applies to auditory processing. Methods: Specifically, this study combined assessments of cognitive functions, an AX discrimination task, and electroencephalography (EEG) to investigate the discrimination results and real-time processing characteristics of native Macau Cantonese speakers perceiving three pairs of similar tones. Results: The behavioral results confirmed the completed merging of T2–T5 in Macau Cantonese, and the ongoing merging of T3–T6 and T4–T6, with perceptual merging rates of 45.46% and 27.28%, respectively. Mismatch negativity (MMN) results from the passive oddball experiment revealed distinct temporal processing patterns for the three tone pairs. Cognitive functions, particularly attention and working memory, significantly influenced tone discrimination, with more pronounced effects observed in the mean amplitude of MMN during T4–T6 discrimination. Differences in MMN peak latency between T3–T6 and T4–T6 further suggested the use of different perceptual strategies for these contour-related tones. Specifically, the T3–T6 pair can be perceived through early signal input, whereas the perception of T4–T6 relies on constant signal input. Conclusions: This distinction in cognitive resource allocation may explain the different merging rates of the two tone pairs. This study, by focusing on the perceptual difficulty of tone pairs and employing EEG techniques, revealed the temporal processing of similar tones by native speakers, providing new insights into tone phoneme processing and speech variation.

## 1. Introduction

Within the framework of Information Processing Theory (IPT), memory is essential for storing and processing information [1,2]. This study specifically investigated how real-time phonological processing, especially in tonal languages like Cantonese, develops the current understanding of IPT by examining the perceptual merging of similar tones. Numerous theories have been proposed to explain short-term or working memory, such as chunking theory [3], the multi-stage model [4], and the working memory model [5]. These models primarily examine how short-term memory facilitates the processing of complex tasks. Theories of working memory have demonstrated considerable explanatory power in various linguistic domains, including syntactic processing (e.g., [6,7,8]), semantic processing (e.g., [9,10,11]), and phonological processing (e.g., [12,13,14]).

Notably, the relationship between phonological processing and working memory has been identified as highly interactive. For instance, previous research observed that deficits in phonological storage within working memory could underlie poor memory performance in children with language disorders, potentially contributing to impaired language development [15]. Lerousseau et al. recently found that working memory deficits in children with cochlear implants are likely due to impairments in the processing, encoding, and storage of auditory and lexical information [16]. In typical populations, individual differences in working memory capacity could influence phonological processing. Zahler and Lord, in a bilingual study, found that Spanish learners with higher phonological short-term memory produced vowels more closely resembling those of native speakers [17]. However, research on the real-time processing of suprasegmental information remains relatively limited. Pitch contours, as a key aspect of suprasegmental information, involve dynamic and continuous acoustic signals (e.g., fundamental frequency, F_0_) that evolve over time. Within the framework of IPT, the processes of encoding, storage, and retrieval in working memory play a crucial role in managing these time-varying signals. Unlike discrete phonemes, pitch contours require listeners to maintain and integrate auditory information over extended periods, relying heavily on the phonological loop in working memory to construct mental representations [18]. This cognitive demand is particularly pronounced in tonal languages like Mandarin and Thai. Studies on tone processing have also demonstrated a positive correlation between working memory capacity and the ability to identify Mandarin tones in noisy environments among Chinese preschoolers aged 5 to 6 [19]. Thai, as another typical tonal language, features three level tones and two contour tones [20]. While few studies directly explore the cognitive demands of Thai tone processing by native speakers, insights can be drawn from studies on non-native speakers. For instance, Mandarin and Vietnamese speakers unfamiliar with Thai performed better in Thai tone imitation under low memory load conditions, with accuracy declining as memory load increased [21]. Similarly, working memory training could enhance speech perception, particularly in noisy environments [22]. In order to explain the mechanism of working memory in speech processing, some scholars argued that effective storage and processing of continuous sound signals are necessary for accurate speech decoding [23,24,25,26]. These studies underscored the crucial role of phonological short-term memory or working memory in facilitating the accurate perception and production of speech sounds.

While previous research has established the link between working memory and phonological processing, the real-time processing of tonal information remains relatively underexplored. Cantonese, with its more complex tonal system compared to Mandarin and Thai, provides a compelling case for investigating the dynamic interaction between cognitive mechanisms and tone perception. This study aimed to address this gap by examining how native Cantonese speakers perceive similar tone pairs during real-time auditory processing. By using neuroscientific tools such as electroencephalography (EEG), known for its high temporal resolution, and mismatch negativity (MMN), a component reflecting short-term memory operations, our findings could offer new insights into real-time auditory processing and contribute to a broader understanding of the feasibility and validity of IPT.

Modern Cantonese is a typical tonal language, predominantly spoken in the Pearl River Delta region of southern China. Within this region, variation exists in the number of tones across different Cantonese dialects. For example, Guangzhou Cantonese, which serves as a representative Cantonese, features nine distinct tones that differentiate lexical meanings [27]. These nine tones vary in pitch or pitch contour, enabling native speakers to distinguish lexical meanings, a primary function of lexical tones [28]. Three of these tones are distinguished by duration and are known as checked tones (i.e., T7, T8, and T9), which end in a consonant and have a shorter duration compared to the other six unchecked tones. Since this paper primarily focuses on the temporal processing of unchecked tones, the term “lexical tones” will hereafter specifically refer to these six unchecked tones. In this study, we use T1–T6 to represent these six lexical tones (cf. Figure 1 of [29] and Figure 2 of this paper). The tonal system of Macau Cantonese is almost identical to that of Guangzhou Cantonese, and both are classified under the Guangfu (廣府, meaning Guangzhou area) subgroup of Cantonese [30,31]. Consequently, this paper used the Guangfu tonal system to investigate the tone perception among native speakers of Macau Cantonese.

The lexical tones in Cantonese, with the exception of T1 [pitch: 55], which is relatively isolated in the upper tonal space, cluster in the mid-to-lower tonal space. This clustering leads some native speakers to confuse these tones [29]. Specifically, T3 [pitch: 33] and T6 [pitch: 22] are level tones that differ only in pitch. T2 [pitch: 25] and T5 [pitch: 23] both have rising contours, yet T2 reaches the highest pitch level, while T5 exhibits only a slight rise. Similarly, T4 [pitch: 21] and T6 [pitch: 22] occupy the lowest tonal space and differ only slightly in contour (ibid.). Furthermore, some native speakers exhibit a compressed tonal space when producing these tones, and some exhibit a longer processing time for the perception of these three tone pairs (i.e., T2–T5, T3–T6, and T4–T6). The findings suggested an ongoing tone merging in Cantonese.

Subsequent studies have extensively explored the merging of these tone pairs. For instance, Ou et al. identified two groups in Hong Kong: individuals with good perception but poor production in T2–T5, and those with poor perception but good production in T4–T6. These groups required significantly more time to distinguish tones compared to those who had not experienced tone merging [25]. In the context of Macau, scholars discovered that T2–T5 has completely merged, while T3–T6 and T4–T6 are still in the process of merging, with the T3–T6 merging being more prevalent [32,33]. This indicated a variation in the merging speed among the three tone pairs.

Wang et al. also examined tone perception and merging in Macau Cantonese. Their study revealed that young speakers in Macau have completed the perceptual merging of T2–T5, while the perceptual merging of T3–T6 and T4–T6 is occurring at a similar rate [26]. The finding that T4–T6 merges more slowly is primarily due to the higher degree of distinction still maintained in production. Thus, the authors suggested that tone merging likely begins with perceptual merging, which, over time, influences production and subsequently spreads within the speech community. However, it should be noted that the reason why T3–T6 and T4–T6, despite their differences in pitch contour, show similar degrees of perceptual merging remains unclear.

Furthermore, these studies have primarily focused on identifying tone merging within speech communities and describing the differences in tone processing among individuals with varying merging patterns, lacking an examination of native speakers’ ability to distinguish different tones. Specifically, it remains unclear whether native speakers, as a healthy and typical group, experience differences in cognitive operation and neural resource allocation when processing different tone pairs. These challenges could potentially lead to differences in the speed and manner of tone merging.

However, although some studies (e.g., [25,26]) have introduced a cognitive perspective and an individual-differences approach, yielding valuable insights, such as the positive role of attention and working memory in tone perception and production, these findings were predominantly based on behavioral results. Importantly, tone perception is an incremental process [34], which requires online processing strategies and mental efforts. Behavioral experiments may not adequately capture the sub-processes and nuanced dynamics involved, as they rely solely on discrimination outcomes. Although Ou and Law examined the real-time processing of Cantonese tones in native Hong Kong speakers using EEG and found that attention and working memory facilitated tone discrimination [24], their study focused exclusively on the T2–T5 tone pair. Our understanding of the processing of T3–T6 and T4–T6 tone pairs remains limited. It is thus essential to explore the real-time processing and specific time courses of tone perception from an online perspective and in light of direct physiological evidence.

In particular, the high temporal resolution offered by EEG and event-related potential (ERP) techniques provides a promising avenue for such investigations. In the realm of auditory perception, research on MMN is highly prevalent, which provides a novel window into central auditory perception and the shaping of auditory memory, allowing for exploration of the underlying neurobiological mechanisms (see review in [35]). MMN is an ERP component related to auditory processing that is obtained by subtracting the response to a standard stimulus from the response to a deviant stimulus [36], necessitating a sequence of sounds where a standard stimulus frequently occurs and a deviant stimulus occurs less frequently. The frequent stimuli form a short-term memory trace in the auditory cortex. When the less frequent sound appears, MMN is elicited if the listener detects a mismatch with the memory trace, even in the absence of voluntary attention. Thus, MMN serves as a measure of automatic detection of acoustic changes [35,37,38,39]. Moreover, significant MMN responses can be elicited by any acoustic change [40].

Law et al. applied MMN technology to the study of T4–T6 perception in Hong Kong Cantonese, finding no significant MMN component in individuals who had merged T4–T6 perceptually [41]. This confirmed that these individuals could not automatically detect the difference between the two tones. They also compared lexical and non-lexical conditions, suggesting that tone recognition involves not only bottom-up analysis but also top-down prediction of acoustic signals. Similar results were obtained for the T2–T5 perceptual merging, with Ou and Law [24,42] also observing a lack of MMN, indicating a perceptual confusion.

Additionally, MMN has been used to detect categorical perception boundaries of tones. For example, there would be no clear perceptual boundary exists between tones with similar contours, while reliable MMN components were observed for tone pairs with saliant contour differences [43]. However, these studies generally focused on contours as a whole and the ability to distinguish between two sounds, rather than examining the temporal progression of contour processing. Empirical research on the processes and constraints involved in sound classification is limited [44].

Kristine provided new insights into the temporal resolution of Cantonese tone processing [45]. In this study, the author adjusted the temporal resolution of F_0_ contours for different tones. For example, the F_0_ contour of the T3 tone in “畏” (/wai/, fear) was divided into segments of 30.41 ms in length (e.g., seven, five, three, or two segments), with the remaining parts filled with white noise (cf. Figure 1 of [45]). Participants were required to identify the sound /wai/ under different temporal resolution conditions. The results showed that while reduced temporal resolution had limited impact on tone recognition overall, the rising tones T2 and T5 were more affected than other tones due to the loss of subtle contrast information. Moreover, Yu noted that the distinction among the four “same-start tones” (i.e., T2, T5, T4, and T6) in Cantonese relies not on contour or starting pitch but on endpoint pitch [46]. Therefore, even with reduced temporal resolution, correct tone identification is possible if endpoint pitch information is preserved. Thus, although native speakers can rely on alternative distinguishing features to identify tones in experimental settings with partial F_0_ information, this might be an adaptive behavior in such contexts. In natural speech conditions, the online processing of different tones by native speakers still remains unclear. More importantly, the cognitive difficulty and real-time processing of different tone pairs by native speakers, and how these factors relate to the process of tone merging, warrant further investigation.

In the current study, we employed an active AX discrimination task alongside a passive oddball task with simultaneous EEG recording to examine the behavior and neural resource allocation of Macau Cantonese speakers when processing different tone pairs (i.e., T2–T5, T3–T6, and T4–T6). Additionally, we administered cognitive function tests, including assessments of attention and working memory, to explore potential correlations between individual cognitive abilities and both behavioral performance and neural activities. The AX discrimination task was designed to assess the encoding and retrieval processes by requiring participants to temporarily retain tonal information and compare it with subsequent auditory input, reflecting the phonological loop’s role in working memory. In contrast, the passive oddball task employed MMN analysis to examine the automatic encoding and storage of auditory stimuli. MMN captures the brain’s ability to detect deviations in auditory input based on stored memory traces, providing insights into the automatic encoding and maintenance of tonal differences in short-term memory. Together, these tasks enabled a comprehensive investigation of the cognitive mechanisms underlying real-time tone perception. Specifically, the study aimed to address the following questions:

1. Do Macau Cantonese speakers differ in behavioral results and MMN responses when processing the three tone pairs?
2. Can the behavioral results and MMN responses of processing different tone pairs reflect the merging process of tone perception in Cantonese?
3. Is there a relationship between the attention and working memory performance of Macau Cantonese speakers and their tone discrimination abilities?

Given that the elicitation of MMN has been widely demonstrated to be associated with various acoustic parameters and sensitive to stimulus deviations of acoustic signals [35], such as pitch [47,48], intensity [49,50], and duration [51,52], it can be effectively used as a measure for assessing native speakers’ perceptual ability of distinguishing Cantonese tone pairs. Notably, Tsang et al. conducted an MMN experiment on four Cantonese tone pairs (i.e., T6–T1, T6–T3, T1–T2, and T6–T2) in Hong Kong Cantonese and found that tone pairs with larger pitch differences elicited greater MMN amplitudes and shorter latencies. Moreover, T1–T2 elicited a greater MMN amplitude and shorter latency than T6–T2, as the former exhibited pitch differences earlier in the contour [53]. Based on this line of evidence and the findings on the tone merging in Macau Cantonese [26], we expect the lowest accuracy and the longest reaction time for T2–T5 in the behavioral data, with no significant differences between T3–T6 and T4–T6. Corresponding to the behavioral results, the ERP results should show no MMN for T2–T5, while significant MMN components are expected for T3–T6 and T4–T6. Additionally, T3–T6 is anticipated to have a larger amplitude and shorter latency compared to T4–T6. Moreover, we anticipate that cognitive functions will not exhibit significant correlations with the behavioral and neural data for the T2–T5 pair. On the contrary, we expect that the correlations observed for the T3–T6 pair will be stronger than those for the T4–T6 pair. The goal of this study is not only to reveal the different temporal perceptual strategies used by Cantonese native speakers to distinguish similar tones but also to provide valuable insights into the suprasegmental processing of tonal languages, and issues related to speech variation. More broadly, our findings can enhance the understanding of sound signal decoding and contribute to the theoretical framework of IPT in general.

## 2. Methods

### 2.1. Participants

A priori power analysis was conducted using G*Power [54] to estimate the required sample size for a repeated-measures ANOVA with within-subject factors. Assuming a medium effect size (f = 0.25), an α-level of 0.05, and a desired power of 0.80, the analysis determined that 28 participants would be necessary to detect significant effects. Based on this calculation, a total of 38 native Cantonese speakers, who were born and raised in Macau, were recruited for this study. Due to data quality issues, 33 participants (18 females) were included in the behavioral analysis while 30 participants (17 females) were included in the EEG analysis. All participants’ ages ranged from 18 to 25 years. The mean age of participants in the behavioral analysis was 20.39 years (*SD* = 2.30), whereas the mean age of participants in the EEG analysis was 20.14 years (*SD* = 2.27). At least one parent of each participant was born and raised in Macau, and Cantonese was the sole language used for family communication. None of the participants had been away from Macau for extended periods in the three years preceding the study. Additionally, all participants were right-handed, reported no hearing or vocal disorders, and were free from mental or neurodegenerative diseases. The study received approval from the local ethics committee, and all participants provided informed consent prior to experiments and received monetary compensation afterward.

### 2.2. Materials

Following the study by Wang et al. [26], the current study employed four CV roots ([si], [ji], [fu], and [se]), combined with six lexical tones, to create various tone pairs (e.g., [si1]/[si1] as an AA pair and [si1]/[si3] as an AB pair) for the AX discrimination task. In other words, the two stimuli in the AA pairs were totally identical, while the two stimuli in the AB pairing differed only in pitch. Ultimately, 32 tone pairs (20 AA pairs and 12 AB pairs) associated with tone merging were designated as test pairs, whereas 16 tone pairs (4 AA pairs and 12 AB pairs) were utilized as fillers, as no merging occurred among them (cf. Table 2 of [26]). In total, the stimuli contained 24 AA pairs and 24 AB pairs. This design aimed to prevent participants from engaging in strategic responses. The materials were recorded from a young female native speaker in Macau using an OLYMPUS LS-100 portable recorder (Olympus Corporation, Tokyo, Japan) and an AKG C-420 microphone (Austrian Kletzsch Gmbh, Vienna, Austria), with a sampling rate of 44,100 Hz and a bit depth of 24 bits. All materials were normalized for F_0_, duration (500 ms), and intensity (70 dB).

Meanwhile, the CV syllable [ji] was selected for the passive oddball task to derive the tone pairs. The selection of [ji] was made to minimize the potential interference from consonant phonemes. Consequently, this study established three conditions: [ji2]/[ji5], [ji3]/[ji6], and [ji4]/[ji6]. In each condition, the first tone served as the standard stimulus, and the second tone served as the deviant stimulus. Each standard stimulus appeared 250 times (83.33%), while the deviant stimulus appeared 50 times (16.67%) in a pseudorandom sequence. Specifically, the first ten stimuli in each sequence were guaranteed to be standard [55], with a minimum of three standard stimuli between every two deviant stimuli. The audio materials used in this experiment were consistent with those in the AX discrimination task.

### 2.3. Procedure

Participants completed all experiments in a soundproof EEG laboratory at the local university. The AX discrimination task was employed to measure participants’ ability to distinguish different tone pairs under active attention, and it was conducted at least one month prior to the passive oddball task to prevent any priming effects. Briefly, this task required participants to wear headphones while AA or AB pairs were randomly presented (for a total of 10 times). Participants were instructed to press a key on the keyboard to determine whether the sounds they heard were the same or different. Each trial began with a 300 ms fixation, followed by the first sound of the pairs. After a 500 ms interval of silence, the second sound was played. Participants could press the key while the second sound was playing. If there was no response within three seconds, the trial was marked as incorrect, and the experiment proceeded to the next trial. After each trial, a randomly varying inter-trial interval of 800 to 1000 ms was implemented. In this paradigm, the discrimination rate and reaction time were obtained for each tone pair for every participant.

Immediately following the completion of the AX discrimination task, participants underwent attention and working memory tests. All tests were conducted using published methods which have demonstrated strong validity in measuring attention and working memory. Specifically, attention was assessed using the Test of Everyday Attention [56], whereas working memory was evaluated using the Digit Span Test from the Wechsler Adult Intelligence Scale, Fourth Edition (WAIS-IV, [57]). Only the subtests related to the auditory modality were employed to evaluate participants’ auditory attention and working memory performance. Scores were assigned based on the manuals, yielding total scores for both attention and working memory.

For the passive oddball task, participants sat comfortably in front of a computer screen, watching a subtitled but silent documentary, while the three oddball conditions were played through in-ear headphones. They were instructed to minimize physical movement and were informed that they would answer questions related to the documentary after it concluded, which was intended to engage participants’ attention on the video. Once the EEG signals were stable, the sound sequences for the three oddball conditions were played. The order of the conditions was randomized. Each stimulus lasted for 500 ms, followed by a 600 ms interval of silence before the next stimulus. A 20 s interval was implemented between two consecutive conditions. All the experimental programs were developed using E-Prime 3.0 (ver: 3.0.3.80, Psychology Software Tools, Pittsburgh, PA, USA).

### 2.4. EEG Recording and Analysis

**Figure 1:**
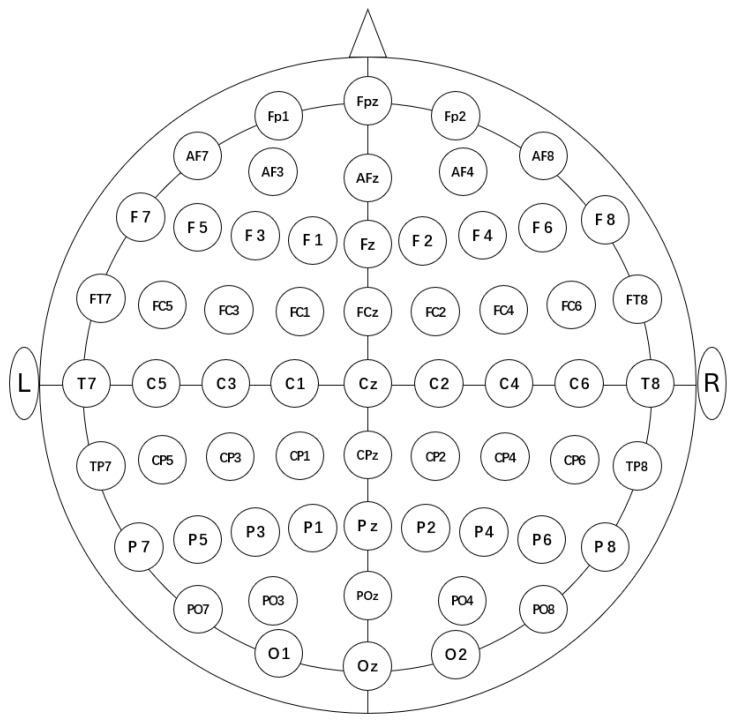
The electrode layout used in this study.

EEG signals were recorded using ANT Neuro’s eego mylab system (EE-225, ANT Neuro, Hengelo, The Netherlands) while participants were performing the oddball task. Participants wore a 64-channel Waveguard Original EEG cap (CA-208, ANT Neuro, The Netherlands) with electrodes arranged according to the 10-20 international system (Figure 1). The reference electrode was placed at CPz and the ground electrode was positioned at AFz. The online sampling rate was set to 1000 Hz, and the electrode impedance for each channel was maintained below 20 kΩ.

EEG signals were preprocessed offline using the EEGLAB toolbox [58]. Signals were re-referenced to the grand average and band-pass filtered between 1 and 20 Hz. Channels with high impedance were identified using automatic bad channel detection and replaced using the spherical interpolation method with data from surrounding electrodes. Independent component analysis (ICA) was performed to remove vertical and horizontal eye artifacts. Subsequently, all signals were time-locked to stimulus onset and segmented into epochs ranging from −200 ms to 800 ms. Epochs with voltage exceeding ±120 µV were excluded, resulting in the rejection of 1035 trials, which constitutes 3.83% of the total trials.

**Figure 2:**
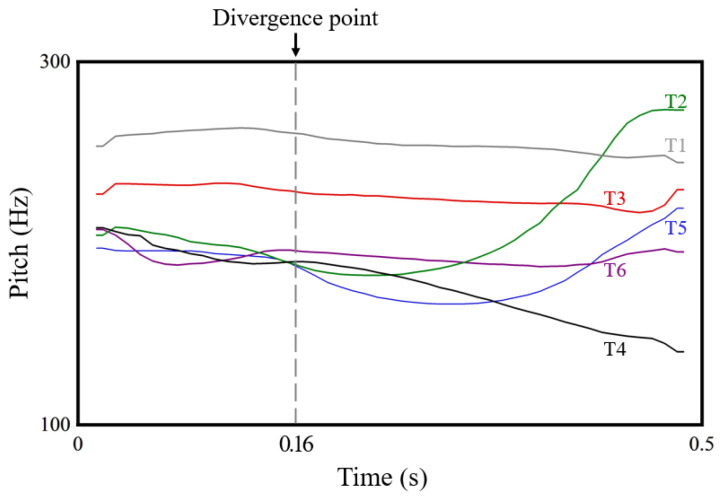
The stimuli based on Guangfu Cantonese tonal system used in this study. The dashed line indicates the divergence point (160 ms) of T2–T5 and T4–T6.

For ERP analysis, the first significant negative deflection following the divergence point was selected as the MMN recognition window to identify potential MMN components. Specifically, the divergence points for T2–T5 and T4–T6 were defined as 160 ms after stimulus onset, as these tone pairs shared similar contours during the initial stage. For T3–T6, the stimulus onset was regarded the divergence point due to the immediate pitch difference (see Figure 2). Given that MMN typically exhibits the maximum response at the frontocentral region [35,59], and based on previous studies [24], the mean amplitude, peak amplitude, and peak latency of MMN were computed at the Fz and FCz electrodes for all trails of each participant under three conditions. The peak amplitude and peak latency were determined based on the most negative peak within the specified time window. Regarding the specific computation of MMN, this study categorized all remaining trials into two types: deviant and standard stimuli. First, the average ERP elicited by the standard stimuli for each participant was computed. Subsequently, this average ERP was subtracted from ERP elicited by each deviant stimulus to obtain individual-specific difference waves. These difference waves were then averaged at both individual and group levels.

### 2.5. Data Analysis

For the features of difference waves obtained from EEG (i.e., mean amplitude, peak amplitude, and peak latency), this study first eliminated trials with values exceeding 2.5 standard deviations (*SD*) for each participant. Subsequently, for the discrimination rate and reaction time data obtained from the AX discrimination task, as well as the attention and working memory scores, we performed logarithmic transformations to normalize the distributions [60]. Given the presence of zero values in these datasets, the logarithmic transformation was conducted after a constant shift (i.e., adding 0.01 to the original values in this study [61]). Since amplitude data included negative values, the treatment of ERP data involved a two-sided logarithmic transformation: the absolute values of the negative values were taken prior to logarithmic transformation, and then these values were restored to negative [62]. For discrimination rate, reaction time, mean amplitude, peak amplitude, and peak latency, we conducted one-way repeated measures ANOVAs to compare the differences in behavior and neural activities of participants when presented with different tone pairs. All post hoc paired comparisons were adjusted using the Benjamini-Hochberg correction (BH, [63]) to control the false discovery rate (FDR), with all subsequent p-values reported as adjusted. To explore the correlations among these indices and cognitive functions, we performed Pearson correlation analyses between the scores of attention/working memory and the behavioral/neural data to identify potential associations.

## 3. Results

### 3.1. Behavioral Results

In the current study, the number of participants who experienced perceptual merging across the three tone pairs was first analyzed based on the AX discrimination task. Following the perceptual merging threshold set in previous research (i.e., 95%, [26,42]), the results showed that T2–T5 exhibited a completed merging among the participants, with all 33 participants unable to perceptually distinguish the two tones. In contrast, for T3–T6 and T4–T6, only 15 and 9 participants, respectively, demonstrated perceptual merging (see Table 1).

**Figure 3:**
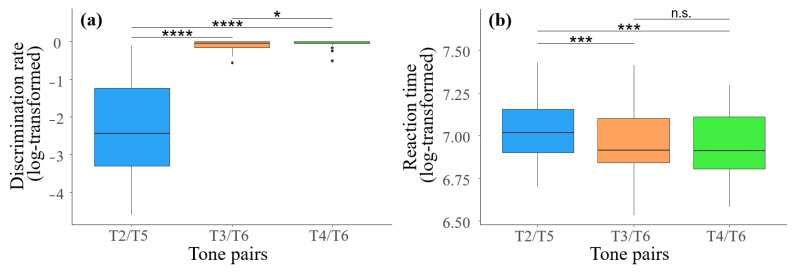
The discrimination rate and reaction time for different tone pairs. (**a**) shows the discrimination rate for different tone pairs; (**b**) shows the reaction times required for recognizing different tone pairs. * denotes *p* < 0.05, *** denotes *p* < 0.001, **** denotes *p* < 0.0001, “n.s.” denotes “not significant”.

The discrimination rate and reaction time for native speakers encountering the three tone pairs are presented in Table 2. The results of a one-way repeated measures ANOVA revealed a significant main effect of tone pair on discrimination rate, *F*(2, 93) = 24.89, *p* < 0.001, *η_p_*^2^ = 0.35, indicating that native speakers’ ability to discriminate different tone pairs varied significantly. Pairwise comparisons indicated that the discrimination rate for T2–T5 (−2.48 ± 1.33) was significantly lower than that for both T3–T6 (−0.09 ± 0.13) and T4–T6 (−0.05 ± 0.10) (*p*s < 0.001). Additionally, the discrimination rate for T3–T6 (−0.09 ± 0.13) was significantly lower than that for T4–T6 (−0.05 ± 0.10) (*p* = 0.02). This indicated that native speakers exhibited the greatest ability to discriminate T4–T6, followed by T3–T6, with the weakest discrimination ability for T2–T5 (Figure 3a).

Regarding reaction time, there was no significant main effect of tone pair, *F*(2, 93) = 0.21, *p* = 0.81, *η_p_*^2^ < 0.01, suggesting no overall significant differences among tone pairs. Although the overall effect was not statistically significant, post hoc comparisons indicated significant differences among certain tone pairs. Pairwise comparisons indicated that the reaction time for T2–T5 (7.04 ± 0.20) was significantly longer than that for both T3–T6 (6.95 ± 0.20) and T4–T6 (6.94 ± 0.18) (*p*s < 0.001). However, there was no significant difference in reaction time between T3–T6 and T4–T6 (*p* = 0.53). This suggested that native speakers require more effort to discriminate T2–T5, whereas discriminating T4–T6 has no obvious advantage compared to T3–T6 (Figure 3b).

### 3.2. ERP Results

**Figure 4:**
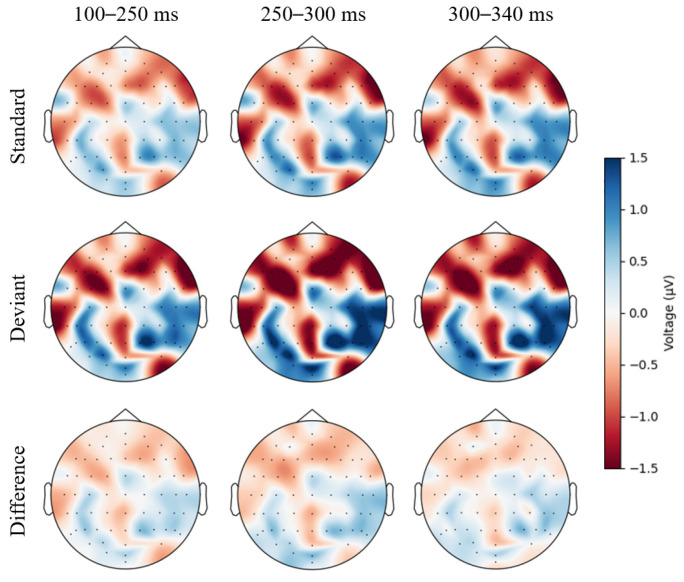
The brain topographic maps of standard stimulus, deviant stimulus, and difference wave in the time windows of 100–250 ms, 250–300 ms, and 300–340 ms under T4–T6 condition.

The grand average ERP waveforms for the three tone pairs recorded at the Fz and FCz electrodes are presented in Figure 4. These waveforms include the ERP responses to both standard and deviant stimuli, as well as the difference waves obtained by subtracting the standard from the deviant.

**Figure 5:**
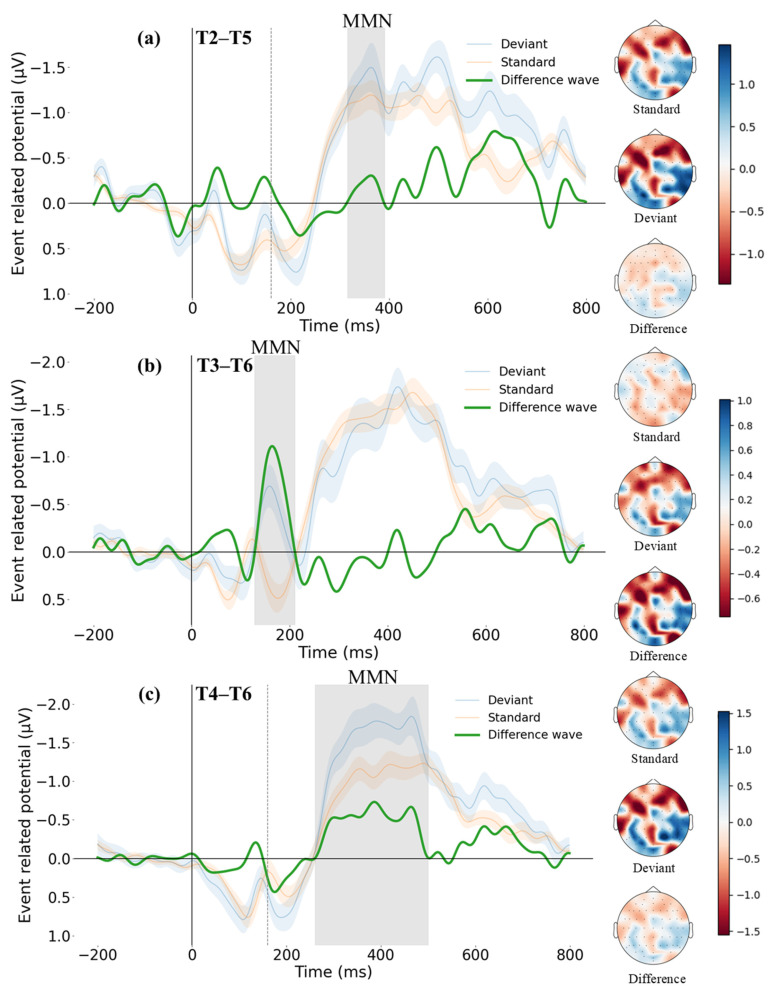
The grand average ERP waveforms at the Fz and FCz electrodes under three conditions, including the standard wave, deviant wave, and difference wave. The topographic maps on the right side of each sub-figure represent, from top to bottom, the standard stimulus, the deviant stimulus, and the difference between them within the respective time window. (**a**) shows the ERP waveforms and topographic maps under the T2–T5 condition; (**b**) shows the ERP waveforms and topographic maps under the T3–T6 condition; (**c**) shows the ERP waveforms and topographic maps under the T4–T6 condition.

To identify significant MMN components, this study defined the presence of prominent negative deflections in the difference waves occurring approximately 250 ms after the divergence point (e.g., [64,65,66]). For the T2–T5 pair, a negative wave, potentially representing the MMN, was observed between 155 and 230 ms after the divergence point. For T3–T6, a significant negative wave was observed between 128 and 210 ms post-stimulus. For T4–T6, the potential MMN time window was extended to 100–340 ms after the divergence point, given that the difference wave voltage for this condition demonstrated an increasing trend beyond the typical MMN window. To ensure that the ERP signal within the time window was not contaminated by other components (e.g., the P3a immediately following it), the long time window was divided into three shorter periods: 100–250 ms (a typical MMN window), 250–300 ms, and 300–340 ms. Topographic maps for these periods were analyzed to identify any distinct activation patterns in the later windows. Visual inspection revealed a consistent frontal negativity distribution across all three shorter windows, indicating stability throughout the 100–340 ms interval. Additionally, comparisons across conditions (T3–T6 and T2–T5; see Figure 5) showed no evidence of specific positive enhancements in the T4–T6 condition, verifying the finding that this window primarily reflects MMN activity rather than contributions from other components.

Subsequently, paired two-sided t-tests were performed on the voltages of standard and deviant stimuli within the identified time windows to validate the identification results [67]. Results indicated that for T2–T5, the deviant stimuli (−1.32 ± 0.13 μV) elicited significantly more negative voltages than the standard stimuli (−1.14 ± 0.04 μV), *t*(75) = −17.55, *p* < 0.001. For T3–T6, deviant stimuli (−0.38 ± 0.24 μV) also produced significantly more negative voltages compared to standard stimuli (0.29 ± 0.18 μV), *t*(82) = −16.99, *p* < 0.001. For T4–T6, the deviant stimuli (−1.49 ± 0.40 μV) elicited significantly more negative voltages than the standard stimuli (−1.00 ± 0.29 μV), *t*(240) = −42.94, *p* < 0.001.

In summary, MMN components were identified for all three tone pairs, although their characteristics varied significantly, as illustrated in Figure 5.

**Figure 6:**
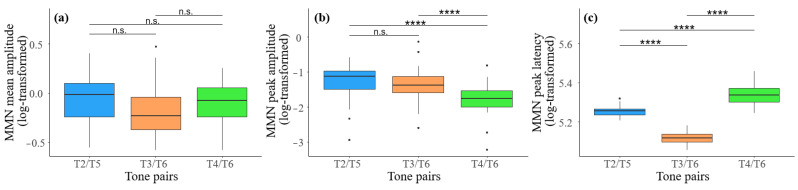
Differences in the mean amplitude, peak amplitude, and peak latency of MMN across the three conditions. (**a**) shows the differences in mean amplitude across the three tone pairs; (**b**) shows the differences in peak amplitude across the three tone pairs; (**c**) shows the differences in peak latency across the three tone pairs. **** denotes *p* < 0.0001, “n.s.” denotes “not significant”.

To compare the three tone pairs regarding differences in mean amplitude, peak amplitude, and peak latency within the respective time windows, we conducted a one-way repeated measures ANOVA for each indicator (see Figure 6).

For mean amplitude, there was no significant main effect of tone pair, *F*(2, 84) = 0.18, *p* = 0.84, *η_p_*^2^ = 0.005. Post hoc pairwise comparisons revealed no significant differences between any of the tone pairs, indicating that the mean amplitudes generated within the time window did not differ across the different tone pairs. Regarding peak amplitude, no significant main effect of tone pair was observed, *F*(2, 84) = 1.48, *p* = 0.23, *η_p_*^2^ = 0.10. However, significant pairwise differences were revealed in the post hoc analysis. The peak amplitude elicited by the T4–T6 pair (−1.80 ± 0.46) was significantly more negative compared to both the T2–T5 (−1.27 ± 0.51) and T3–T6 (−1.38 ± 0.48) pairs, with *p*s < 0.001. Meanwhile, there was no significant difference between the T2–T5 (−1.27 ± 0.51) and T3–T6 (1.38 ± 0.48) pairs (*p* = 0.56). For peak latency, a significant main effect of tone pair was found, *F*(2, 84) = 59.92, *p* < 0.001, *η_p_*^2^ = 0.59. Pairwise comparisons indicated that the peak latency for T4–T6 (5.34 ± 0.05) was significantly later than both T3–T6 (5.12 ± 0.03) and T2–T5 (5.25 ± 0.03), with *p*s < 0.001. The latency of T3–T6 (5.12 ± 0.03) was significantly shorter than that of T2–T5 (5.25 ± 0.03) (*p* < 0.001).

### 3.3. Correlation Analysis

In terms of the correlation between behavioral data and cognitive functions, no significant correlations were found for the T2–T5 pair between the discrimination rate and reaction time with either attention or working memory (*p*s > 0.05) (see Table 3). For the T3–T6 pair, the discrimination rate was moderately positively correlated with both attention and working memory (attention: *r*(28) = 0.41, *p* = 0.03; working memory: *r*(28) = 0.40, *p* = 0.03), indicating that better attention and working memory are associated with an enhanced ability to discriminate the two tones, while no significant associations were observed between reaction time and cognitive functions (*p*s > 0.05) (see Table 4). Similarly, for the T4–T6 pair, a significant positive correlation was found between discrimination rate and attention (*r*(28) = 0.47, *p* = 0.01), yet no significant relationships were detected between reaction time and cognitive functions (*p*s > 0.05) (see Table 5).

Regarding the relationship between neural responses and cognitive functions, no significant correlations were found for the T2–T5 pair concerning mean amplitude, peak amplitude, or peak latency (*p*s > 0.05) (see Table 3). Similar results were observed for the T3–T6 pair as well (*p*s > 0.05) (see Table 4). However, for the T4–T6 pair, attention and working memory exhibited moderate negative correlations with mean amplitude (attention: *r*(28) = −0.41, *p* = 0.03; working memory: *r*(28) = −0.44, *p* = 0.02), indicating that higher levels of attention and working memory were associated with more negative mean amplitudes. No significant relationships were detected for peak amplitude and peak latency (*p*s > 0.05) (see Table 5).

Additionally, this study analyzed the results of the AX discrimination task alongside neural responses. The results indicated that for the T2–T5 pair, there were no significant correlations between the discrimination rate and reaction time concerning the mean amplitude and peak amplitude (*p*s > 0.05) (see Table 3). However, a negative trend was observed between peak latency and discrimination rate (*r*(28) = −0.33, *p* = 0.09), suggesting that longer latency may be associated with lower discrimination rate. In contrast, for the T3–T6 pair, a significant positive correlation was found between peak latency and discrimination rate (*r*(28) = 0.42, *p* = 0.03), indicating that longer latencies were associated with a stronger ability to discriminate the two tones (see Table 4). For T4–T6, the overall discrimination rate was relatively high, but only reaction time showed a significant negative correlation with peak latency (*r*(28) = −0.38, *p* = 0.04), suggesting that longer latencies were associated with quicker discrimination times. No other significant correlations were found in the remaining comparisons (*p*s > 0.05) (see Table 3).

## 4. Discussion

This study investigated the behavioral and neural responses of native Macau Cantonese speakers to similar tone pairs in both active and passive tone perception tasks. Specifically, the study aimed to explore differences in neural resource allocation over time during the processing of these tone pairs, as well as to assess the impact of cognitive functions, including attention and working memory, on tone discrimination abilities. The findings provided new insights into tone contour processing and Cantonese tone variation issues, highlighting the significant role of attention and working memory in facilitating tone perception.

Behaviorally, the perceptual merging results obtained in this study were consistent with those of Wang et al. [26]. In their study, T2–T5 also exhibited a completed merging, while the proportions of perceptual merging for T3–T6 and T4–T6 were 40.91% and 34.10%, respectively, closely aligning with the 45.46% and 27.28% observed in our study. However, the perceptual merging difference between T3–T6 and T4–T6 in our study was slightly larger. We attributed this discrepancy in the merging proportions to the inherent variability in random sampling, which may have influenced the exact percentages observed. Furthermore, our results revealed significant differences in both discrimination rate and reaction time across the three tone pairs. Specifically, the discrimination rate for T2–T5 was significantly lower than that for T3–T6 and T4–T6, which can be clearly attributed to the completed merging of T2–T5. In comparison, T4–T6 had a significantly higher discrimination rate than T3–T6, supporting previous conclusions that the merging process for T4–T6 is the slowest and is currently in the initial stage of merging [26]. However, the finding regarding the slowest merging process for T4–T6 was based on production distinctions in the previous study. In terms of perception, there was no clear advantage for T4–T6 over T3–T6 (cf. Table 3 of [26]). Why does the T4–T6 pair show the most accurate discrimination? One possible reason for this discrepancy is that, in our study, a larger number of participants successfully distinguished the T4–T6 pair, which may have contributed to the higher discrimination rate observed for this tone pair. On the other hand, the reaction time data can also address this question. Aside from the expected longer reaction time for T2–T5 compared to the other two tone pairs, there was no significant difference in reaction time between T3–T6 and T4–T6. The longer reaction time for T4–T6 suggested that although this tone pair has clearer distinguishing features (such as contour and slope), native speakers require more acoustic input to confirm these differences, particularly because the pitch difference emerges gradually, resulting in delayed but more accurate responses. Given that the behavioral results did not fully elucidate the cognitive processes involved before discrimination, we anticipated that ERP findings will provide insights into the real-time processing of these tone pairs.

Using a passive oddball paradigm, our ERP study detected MMN components for all three tone pairs (i.e., T2–T5, T3–T6, and T4–T6), though with differing characteristics. Unexpectedly, an MMN response was observed in the T2–T5 pair, despite previous studies [24,41,42] showing its absence in perceptually merging groups. As highlighted by Shuai and Gong, tone processing involves bottom-up mechanisms that handle pitch contour changes at the acoustic level and top-down mechanisms that govern lexical tone distinctions at the phonological level [68]. Our findings indicated that while T2–T5 tones have merged at the phonological level, Macau native speakers still demonstrated bottom-up processing of pitch contour differences at the acoustic level. This suggested that while the acoustic differences between T2 and T5 have lost their linguistic significance in Macau Cantonese, native speakers retain residual sensitivity to fine-grained pitch differences. Additionally, the shorter inter-stimulus interval (ISI) used in this study (600 ms, shorter than 800 ms in previous studies) likely facilitated efficient comparison of new stimuli with auditory memory traces, enhancing the salience of deviant stimuli and eliciting MMN more robustly.

For the T3–T6 condition, a typical MMN wave was induced after stimulus presentation, with significant activation observed at the frontocentral location (Fz and FCz electrodes), indicating that native speakers retain strong perceptual abilities for cross-categorical tone distinctions. Notably, the MMN identification window for the T4–T6 condition was longer in this study. This extended window was due to the sustained enhancement of the difference wave observed in this period, suggesting ongoing deviation detection by native speakers. Although common research typically defined MMN time windows as between 100 and 250 ms (e.g., [69,70,71]), some studies have indicated that automatic detection of deviations in constant sine wave tones can extend up to 350 ms, beyond which MMN significantly diminishes or disappears [72]. Therefore, the 100–340 ms time window chosen in this study still reflected the automatic detection of differences between the two tones, and it was likely that this reflected an enhancement in native speakers’ detection capabilities for T4–T6 over time.

Further, this study conducted a series of repeated measures ANOVA on the mean amplitude, peak amplitude, and peak latency of MMN across the three tone pair conditions. The results showed an absence of significant differences in mean MMN amplitude among the three tone pairs. These results may be attributed to the way we defined the time window for MMN. In our study, the entire negative deflection was included in the statistical analysis, which may have diluted the ability of mean amplitude to accurately reflect the strength of neural activity. As a result, the mean amplitude differences among the tone pairs were less pronounced. Notably, the current study found that the peak amplitudes for both T3–T6 and T4–T6 were significantly more negative compared to T2–T5, with T4–T6 showing a more negative peak amplitude than T3–T6. This confirmed the degree of perceptual merging of the three tone pairs in the speech community, with T2–T5 having completed merging, T4–T6 in the initial stage of merging, and T3–T6 having a relatively more advanced degree of merging [26]. Regarding peak latency, this study found that the latency for T4–T6 was significantly longer than for T2–T5. Interestingly, the latency for T4–T6 was also notably longer compared to T3–T6. This finding supported our behavioral results, which indicated that native speakers had a longer response time for T4–T6 than expected and required a relatively higher level of certainty to make a discrimination. From the perspective of pitch contour differences between the two tone pairs, the difference between T3 and T6 was apparent from the beginning, allowing for immediate and rapid discrimination by native speakers. In contrast, the pitch contour difference between T4 and T6 gradually expanded after the divergence point. This finding was also consistent with Tsang et al. [53]. They found that the latency was longer for the T6–T2 pair compared to T1–T2, due to the increasing pitch difference between the tones over time. Given the slope difference between T4 and T6, perceiving this difference cannot rely on isolated points but requires continuous acoustic input to build a model of the slope in the mind. Once enough signal input is available, it can be compared with the existing short memory trace in the brain [35], thus enabling the decision of whether the two sounds are consistent.

In terms of the correlation between the AX discrimination task and cognitive functions, our results were largely consistent with previous research [26]. Similarly, we observed no significant correlations between either the discrimination rate or reaction time and cognitive functions for the T2–T5 pair. For the T3–T6 pair, however, there were moderate positive correlations between the discrimination rate and both attention and working memory, indicating that higher levels of attention and greater working memory capacity enhance perceptual discrimination for T3–T6. For T4–T6, only attention showed a moderate positive correlation with the discrimination rate. These findings further corroborated the tone merging process in Macau Cantonese, where T2–T5 has fully merged and is no longer linked to cognitive modulations. In contrast, T3–T6 is undergoing rapid merging and demands more cognitive resources, while T4–T6 is in the initial stage of merging, with a weaker relationship with cognitive functions compared to T3–T6.

Regarding the MMN results, no correlations were found between cognitive functions and the mean amplitude, peak amplitude, or peak latency for the T2–T5 and T3–T6 pairs. However, for T4–T6, the mean amplitude exhibited a moderate negative correlation with both attention and working memory, suggesting that cognitive functions play a significant role in modulating neural responses during tone discrimination. According to models of MMN generation, such as Näätänen’s predictive coding framework [73], MMN reflects the brain’s ability to detect violations of auditory regularities based on stored memory traces. Higher levels of attention and working memory may enhance the encoding of these auditory regularities, leading to stronger neural responses to deviations, as reflected by more negative mean amplitudes. In view of the continuously expanding pitch difference between T4 and T6, the continuous detection of acoustic signals and the active maintenance of tone representations seem to rely on cognitive resources, which contribute to the amplitude patterns observed for T4–T6. These findings shed light on the relationship between neural activities and cognitive functions. The fact that T2–T5 and T3–T6 have comparable amplitudes to T4–T6 but show no correlation with cognitive functions suggested that other factors may influence the passive discrimination of these two tone pairs. These factors could include differences in top-down versus bottom-up processing or task difficulty, which warrant further investigation.

In terms of the correlation between the AX discrimination task and MMN results, no significant correlations were found between the discrimination rate, reaction time, and MMN measures for the T2–T5 pair. This lack of correlation suggested that the generation of MMN for T2–T5 did not stem from the phonological level, as reflected in the AX discrimination task, but rather from the acoustic level, indicating bottom-up processing. For T3–T6, a marginal significant positive correlation was observed between the discrimination rate and peak latency, with longer latencies corresponding to higher discrimination rates. This may reflect the additional cognitive efforts required for pitch distinctions—longer latencies may indicate an extended neural processing time, ultimately leading to more accurate tone discrimination. In contrast, for T4–T6, a significant negative correlation was found between reaction time and peak latency, where longer latency corresponded to a faster reaction time. This finding suggested that for T4–T6, the gradual emergence of pitch differences initially demands greater cognitive effort, leading to delayed neural discrimination, as reflected by longer peak latency. However, once sufficient auditory information is available, participants quickly engage in top-down processing, enabling faster behavioral judgments. This may indicate an efficient integration of sensory evidence with decision-making mechanisms, where the delay in neural discrimination is compensated by accelerated cognitive processing to finalize the response.

Altogether, the results of this study provided both behavioral and electrophysiological evidence for the perceptual merging process of Cantonese tones in Macau. By examining the difficulty of native speakers in distinguishing different tone pairs, we found that the observed ability to differentiate them not only differed but also aligned with the degrees of tone perceptual merging. For T2–T5, the merging phenomenon was reflected in a very low discrimination rate and the longest reaction time. Accordingly, a weaker peak amplitude in MMN was observed under the T2–T5 pair. These indicators objectively showed the difficulty native speakers have in discriminating T2–T5, which we attributed to T2–T5 being perceived as the same phoneme category by native speakers, resulting in decreased sensitivity to acoustic changes within the category [44]. Thus, cognitive functions showed no correlation with either the behavioral or neural measures for the discrimination of the T2–T5 tone pair. The T3–T6 condition elicited a typical MMN component, indicating that native speakers remain sensitive to pitch differences between the two tones. Cognitive functions also significantly facilitated the discrimination of the T3–T6 pair, but they did not affect the MMN responses for it. Our study attributed this to the relatively fast automatic detection of T3–T6, which may not be sensitive enough to reveal the influence of cognitive functions. Compared to T3–T6, even though the mean amplitude for T4–T6 showed a negative correlation with cognitive functions, there were no significant differences in either reaction time or mean amplitude between T4–T6 and T3–T6. This confirmed that the perceptual merging degrees of T4–T6 and T3–T6 are comparable in Cantonese. However, the discrimination rate for T4–T6 was significantly higher than for T3–T6. Through comparisons of peak latency, we suggested that this higher discrimination rate for T4–T6 came at the cost of increased reaction time, as native speakers need to wait for a sufficient acoustic signal input to make a discrimination. This aligned with Yu’s conclusion that T4–T6 relies on endpoint pitch differences [46]. The apparent discrepancy where T4–T6 pair has more distinct features but shows similar perceptual merging to T3–T6 is likely due to this delayed perceptual ability. Based on these findings, we proposed that native speakers employ two different temporal perceptual strategies for distinguishing T3–T6 and T4–T6. For T3–T6, where the tones are almost parallel throughout, native speakers recruit a strategy that employs the very early input signals to differentiate them. In contrast, for T4–T6, where pitch differences gradually emerge after the divergence point, native speakers adopt a strategy that relies on the input of constant acoustic signals. This difference in perceptual strategy results in a certain degree of weakened perception ability for T4–T6, leading to broader perceptual merging.

In conclusion, this study addressed the research questions by demonstrating that native Macau Cantonese speakers exhibit distinct behavioral patterns and cognitive resource allocation when processing the three merging tone pairs (i.e., T2–T5, T3–T6, and T4–T6). These differences mirror the varying degrees of tone merging within the speech community, with cognitive functions like attention and working memory playing a crucial role in tone discrimination. The findings not only enhance our understanding of the tone merging process but also offer new insights into how suprasegmental features are processed within the framework of IPT. By examining the distinctions among tone pairs, we addressed the relationship between the speed of tone merging and general cognitive functions and information processing strategies, offering a valuable perspective for research on tone variation. Moreover, the findings of this study further expanded the current understanding of IPT. We found that even when faced with the same task, variations in the acoustic information received led participants to adopt different processing strategies. This suggested that even within the framework of the working memory model, particularly the phonological loop, individuals can autonomously select different processing methods based on the characteristics of the input. This is likely driven by the need to balance decision speed and accuracy. These insights contributed to refining the Information Processing Theory, particularly in the processing of suprasegmental information.

While this work provided novel insights into the temporal processing of tone contours and the tone merging in the perception of Macau Cantonese, there are additional issues that need to be clarified. As noted in the introduction, our previous conclusion that T4–T6 has the slowest merging rate was based on their high distinguishability in production, which supported the view that perception and production are mediated by distinct brain networks [74]. Nonetheless, this does not mean that we discard the idea of interaction between perception and production; rather, the interaction between the two may require more time. Given the unique position of T4–T6 at the lower end of the tonal space, native speakers might use other cues to aid in discrimination or realization, such as the role of creaky voice in tone perception proposed by Yu and Lam [75] and the finding by Bei and Xiang [30] that T4 has the shortest duration in tone realization. These factors play a significant role in natural speech environments, yet they were not included in our current design. Future research could also explore additional acoustic cues, such as phonation type and duration, to determine how these factors influence the real-time processing of similar tones. Confounding factors should also be considered in the future including individual differences in auditory sensitivity, which can be assessed by more precise metrics such as d-prime (d′) scores, or task familiarity, which may allow participants to develop a rhythmic prediction of the oddball sequence. Additionally, since T2–T5 has already merged in Macau, the perceptual strategies for T2–T5 were not discussed in this study. Interested researchers in other Cantonese regions may further investigate the online processing strategies for T2–T5. Expanding the study to other dialects or age groups could further validate the generalizability of the findings.

## 5. Conclusions

This study provided both behavioral and neural evidence for the perception-based merging of Cantonese tones in Macau, highlighting that the allocation of cognitive resources by native speakers is a crucial factor influencing the tone merging process. Furthermore, by comparing MMN components, this research proposes distinct temporal perceptual strategies for perceiving the T3–T6 and T4–T6 tone pairs: T3–T6 involves a perception process that relies on early acoustic signal input, while T4–T6 employs a perception process that relies on constant acoustic input. The differences in contour-related tone perceptual strategies affect both behavioral performance and the merging process for T4–T6. By integrating behavioral and EEG data, this study not only emphasized the critical role of perceptual strategies in interpreting tone variation but also provided insights into how these strategies contribute to our understanding of tone variation and auditory information processing.

## Figures and Tables

**Table 1:** The number of participants who experienced perceptual merging for each of the three tone pairs (percentages in parentheses).

Tone Pairs	T2–T5	T3–T6	T4–T6
Number of perceptual merging	33 (100%)	15 (45.46%)	9 (27.28%)

**Table 2:** Discrimination rate (%) and reaction time (ms) for the three tone pairs (standard deviations in parentheses).

Tone Pairs	T2–T5	T3–T6	T4–T6
Discrimination rate	16.00 (19.85)	91.11 (10.68)	94.75 (7.87)
Reaction time	1157.80 (227.47)	1063.84 (213.81)	1050.86 (195.09)

**Table 3:** Correlation among behavior, cognitive function, and neural performance for T2–T5 pair.

T2–T5	Discrimination Rate	Reaction Time	Attention	Working Memory	Mean Amplitude	Peak Amplitude	Peak Latency
Reaction time	/						
Attention	−0.06	0.16					
Working memory	−0.21	0.06	/				
Mean amplitude	0.24	0.29	−0.06	0.15			
Peak amplitude	0.08	0.14	−0.02	0.01	/		
Peak latency	−0.33	−0.04	0.10	0.15	/	/	

**Table 4:** Correlation among behavior, cognitive function, and neural performance for T3–T6 pair.

T3–T6	Discrimination Rate	Reaction Time	Attention	Working Memory	Mean Amplitude	Peak Amplitude	Peak Latency
Reaction time	/						
Attention	0.41 *	0.12					
Working memory	0.40 *	−0.04	/				
Mean amplitude	0.19	−0.16	−0.16	0.05			
Peak amplitude	0.16	0.15	0.10	0.20	/		
Peak latency	0.42 *	−0.08	−0.24	−0.12	/	/	

* denotes *p* < 0.05.

**Table 5:** Correlation among behavior, cognitive function, and neural performance for T4–T6 pair.

T4–T6	Discrimination Rate	Reaction Time	Attention	Working Memory	Mean Amplitude	Peak Amplitude	Peak Latency
Reaction time	/						
Attention	0.47 *	−0.07					
Working memory	0.23	−0.15	/				
Mean amplitude	−0.13	0.20	−0.41 *	−0.44 *			
Peak amplitude	0.07	0.04	−0.08	0.01	/		
Peak latency	0.30	−0.38 *	−0.01	0.08	/	/	

* denotes *p* < 0.05.

## Data Availability

The curated data for this study are available at osf.io/et7hy/ (access on 12 December 2024). This contains all the data to support the analysis in the current study. For more detailed raw data, interested teams may contact the corresponding authors with reasonable requests.
